# Immune-checkpoint protein VISTA in allergic, autoimmune disease and transplant rejection

**DOI:** 10.3389/fimmu.2023.1194421

**Published:** 2023-06-26

**Authors:** Meijun Zheng, Zongliang Zhang, Lingyu Yu, Zeng Wang, Yijun Dong, Aiping Tong, Hui Yang

**Affiliations:** ^1^ Department of Otolaryngology, Head and Neck Surgery, West China Hospital, West China Medical School, Sichuan University, Chengdu, Sichuan, China; ^2^ State Key Laboratory of Biotherapy, West China Medical School, Sichuan University, Chengdu, Sichuan, China

**Keywords:** V-domain Ig suppressor of T cell activation, VISTA, allergy, autoimmune disease, transplantation

## Abstract

Negative checkpoint regulators (NCRs) reduce the T cell immune response against self-antigens and limit autoimmune disease development. V-domain Ig suppressor of T cell activation (VISTA), a novel immune checkpoint in the B7 family, has recently been identified as one of the NCRs. VISTA maintains T cell quiescence and peripheral tolerance. VISTA targeting has shown promising results in treating immune-related diseases, including cancer and autoimmune disease. In this review, we summarize and discuss the immunomodulatory role of VISTA, its therapeutic potential in allergic, autoimmune disease, and transplant rejection, as well as the current therapeutic antibodies, to present a new method for regulating immune responses and achieving durable tolerance for the treatment of autoimmune disease and transplantation.

## Introduction

Immune checkpoints inhibitors transmit coinhibitory signals during T cell activation, thereby directly suppressing T cell responses (1). Coinhibitory molecules on T cells, including programmed Death-1 (PD-1), cytotoxic T lymphocyte antigen 4 (CTLA-4), T cell immunoglobulin and mucin domain 3 (Tim-3), Lymphocyte-activation gene 3 (Lag-3), and B and T lymphocyte attenuator (BTLA), can disrupt signaling mechanisms or induce negative intracellular signaling pathways on antigen-presenting cells (APCs) or other cells types (1, 2). Several immune-checkpoint regulator molecules are critical in modulating T cell responses and involved abnormally T cells response mediated inflammatory and autoimmunity diseases (3), which revitalized interest in utilizing antibodies against immunosuppress checkpoint regulators to counter the effects of these molecules.

V-domain immunoglobulin suppressor of T-cell activation (VISTA) is a novel immune inhibitory molecule of the B7 family (4) that can negatively regulate immune responses, maintains peripheral tolerance, and control autoimmunity (5, 6). VISTA is revealed to function as an immune checkpoint in multiple mouse immune pathology diseases models, such as graft-versus-host disease (GVHD), rheumatoid arthritis (RA), asthma, and experimental allergic encephalomyelitis (EAE), and VISTA-deficient mice increase susceptibility to developing autoimmunity (7). The multi-faceted role of VISTA in regulating immune responses has suggested the utility of VISTA as a potential reagent in the prevention or even therapeutic cure of allergic, autoimmune disease and transplant rejection (7–9).

This review aims to illuminate the expression and biological effects of VISTA and discuss the current research progress in preclinical studies on allergies, autoimmune diseases, and organ transplantation using VISTA. In addition, VISTA’s potential as an immune modulator encourages further research on current antibodies, which can control immune responses and achieve durable tolerance for the treatment of autoimmune disease and transplantation.

## The structure and expression of VISTA

VSITA, also known as PD-1H (10), c10orf54 (11), DD1α (12), Dies1 (13), is a type I transmembrane protein on chromosome 10q22.1 containing 311 amino acids (14). VISTA comprises an extracellular IgV domain, a stalk segment, a transmembrane region, and a conserved cytoplasmic tail. The structure of VISTA shares similarities with PD1, CD28, and CTLA4 (15), but the cytoplasmic tail of VISTA lacks a classic immunoreceptor tyrosine-based inhibition motif (ITIM) or immunoreceptor tyrosine-based activation motif (ITAM) (4). VISTA is conserved with 76% identity between humans and mice. Interestingly, the cytoplasmic tail shares 90.6% identity between humans and mice, indicating a highly conserved functional role, whereas the cytoplasmic tail of PD1 between humans and mice only shares 59% identity (4).

VISTA is expressed in several organs and tissues, including the spleen, heart, kidney, thymus, brain, and bone marrow (14). Expression of VISTA is constitutive and limited to hematopoietic cells; there is a significant expression of VISTA in monocytes, macrophages, neutrophils, and dendritic cells (DCs), as well as on all types of T cells, including naïve CD4 and CD8 T cells and regulatory T cells (Tregs), both in human (16) and mice (10, 14), but not on B cells. Interestingly, VISTA is not only expressed on the cell surface, but also expressed in the intracellular of myeloid compartment (17). B lymphocytes, natural killer cells (NK), and other lymphocytes expressed by mice and humans have almost no expression of VISTA (14). VISTA is also expressed in various types of tumors, including ovarian and endometrial cancer (18), prostate cancer (19), hepatocellular carcinomas (20), pancreatic cancer (21), gastric carcinoma (22), and colorectal carcinoma (23). In the tumor microenvironment, VISTA is highly expressed on myeloid-derived suppressor cells (MDSC) and regulates their effector functions (24, 25).

## VISTA binding partners

**Figure 1:**
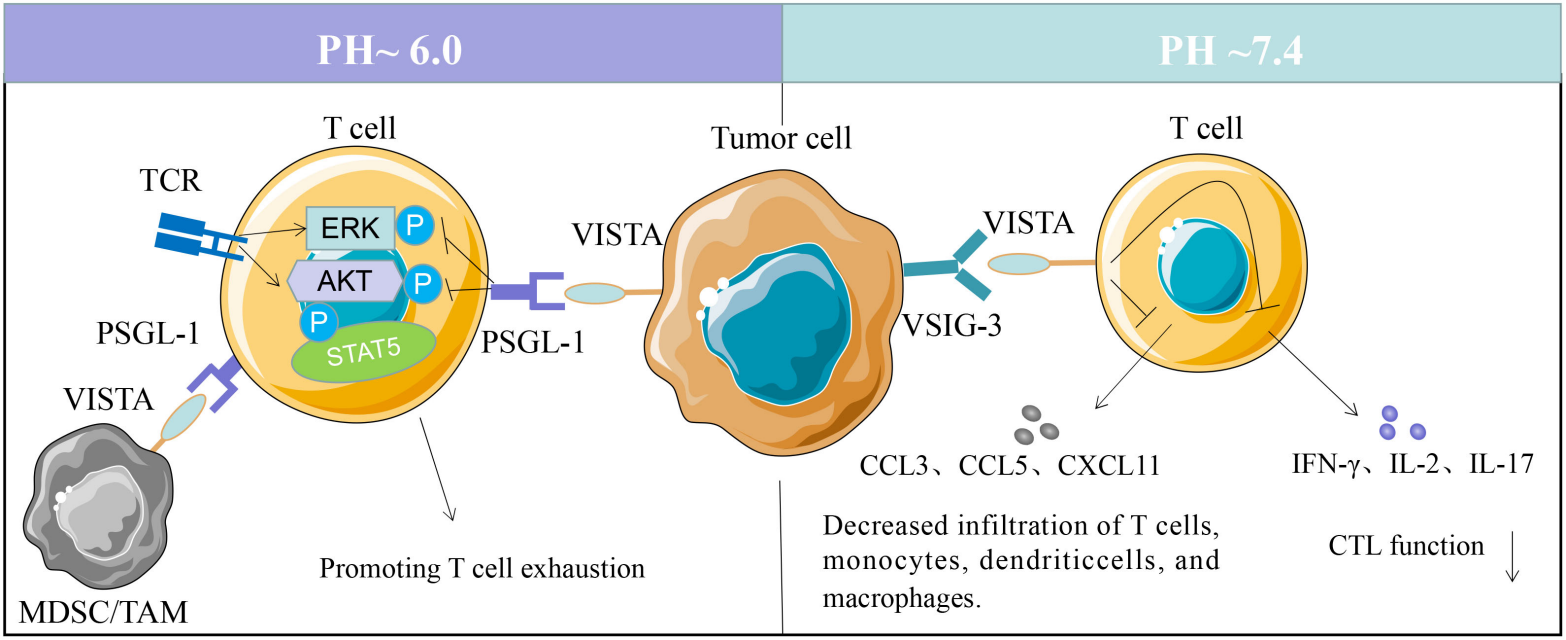
VISTA binding partners. VSIG3 interaction with VISTA on T cells suppresses T cell activation and proliferation. VISTA activity decreases reducing the production of several cytokines, including IFN-γ, IL-2, IL-17, CCL3, CCL5, and CXCL11. VISTA expressed on tumor cells, tumor-associated macrophages (TAMs) or MDSCs can bind to PSGL-1 on T cells at pH 6.0, and promoting T cell exhaustion.

As VISTA contains two potential protein kinase C binding sites in its cytoplasmic tail domain (14), that could function as docking sites, suggesting that it is a coinhibitory molecule with dual functions as both receptor and ligand (11). VISTA can function as a coinhibitory receptor on T cells to suppress naive T cell response. VISTA expressed in APCs interacts with certain T cell receptors to inhibit T cell activation and proliferation (11). Several researchers have identified a potential VISTA-binding ligand, but the counterreceptor of VISTA remains elusive. There are two confirmed ligands of VISTA with immunosuppressive functions; one is VSIG3 (V-Set and Immunoglobulin domain containing 3) and PSGL1 (P-selectin glycoprotein ligand 1) (26, 27). VISTA interacts with VSIG3 at physiological PH environment, while at acidic pH environment to PSGL-1, both interactions led to the suppression of T cell function (26, 27) (**Figure 1**).

Wang and colleagues reported that VSIG3 is a ligand of VISTA that inhibits the proliferation and functions of human T cells. The expression of VSIG3 is primarily observed in non-hematopoietic cells (26). VISTA interacts with VSIG3 *in vitro*, reducing the production of several cytokines, including IFN-γ, IL-2, IL-17, CCL3, CCL5, and CXCL11 (26). However, VSIG3 expression is undetectable in hematopoietic cells, and the physiological relevance of VISTA-VSIG3 interaction *in vivo* remains to be validated. Korman et al. identified PSGL-1 as a VISTA binding partner *via* histidine residues within the extracellular domain (27), and the interaction is regulated by the pH in microenvironment (27). VISTA interacts with PSGL-1 *in vitro* and *in vivo*, suppressing T-cell response at acidic pH 6.0 in the tumor microenvironment (28). Unlike VSIG3, PSGL-1 is expressed on a range of hematopoietic cells, with a lower expression on B cells and microvascular endothelial cells (29, 30). The functional binding of VISTA and PSGL-1 is regulated by tyrosine sulfation and glycosylation (31, 32). PSGL-1 are highly expressed in macrophages, granulocytes, microglia (17), and endothelial cells (28). There is a possibility that VISTA may have multiple binding partners. VSIG8, Galectin 9, and NSC622608 have been reported to bind to VISTA in addition to these two ligands, but further research is required to verify their functional binding (28, 33, 34).

## Roles of VISTA in immunity interactions

Since VISTA is expressed across allergies, autoimmune diseases, and organ transplantation, and therapeutic agents are being developed to target VISTA, it is crucial to determine which cells are affected by VISTA expression.

## Age and gender

VISTA-deficient mice demonstrate an age-related proinflammatory signature (35). VISTA-deficient mice showed autoimmune defects apparent in the aged female mice and an accumulation of anti-nuclear and anti-dsDNA antibodies in the serum, spontaneous glomerulonephritis, and inflammatory phenotypes in the skin, eyes, and ears (12, 35). Interestingly, female animals are substantially more sensitive to the disease in various animal autoimmune disease models, including SLE (36). Similarly, extremely high gender bias (9:1) also has been described in humans for systemic autoimmune diseases (37).

## T lymphocytes

VITSA is essential to maintain peripheral homeostasis of T cells. VISTA-deficient mice exhibit less peripheral T cell deletion and leading to the development of autoimmune phenotypes (5, 7). The transcriptomic analysis of T cells from VISTA-deficient mice demonstrates the importance of VISTA in maintaining naïve T cell quiescence and in activating T cells at early stage (5). Many studies suggest that VISTA on both T cells and APCs negatively regulates T cell responses *via* specific pathways, by acting as a ligand or a receptor. As a receptor on T cells, VISTA can function independently of APCs to negatively regulate T cell responses, as VISTA agonistic suppresses antigen-specific T cell proliferation when activated by VISTA-deficient APCs (2). In addition, VISTA-Ig fusion protein can inhibit T cell proliferation and reduce cytokine production.

Several experimental settings have explored the involvement of VISTA in T helper type 1 (Th1), type 2 (Th2), and type 17 (Th17) mediated immune responses. Most research has demonstrated that VISTA mediates immune responses mediated by Th1 and Th17 cells. Reducing VISTA expression can facilitate the expansion and differentiation of Th1 and Th17 T cells (38). Psoriasiform dermatitis is exacerbated by VISTA deficiency, which increases inflammatory responses in DCs, T cells, and Th17 cells (39). Consistently, murine VISTA knockout models raise susceptibility to develop experimental autoimmune encephalomyelitis that has been reported to be characterized by a Th1/Th17 response (40). The DC in VISTA deficient mice produces more IL-23, which further augments the production of IL17a by Th17 and γδ T cells (40). Compared to wild-type induced Tregs (iTregs), VISTA deficient mice have higher Th17 and Th1 cell formation rates during inflammation, which indicates that VISTA deficient mice have a more reactive T cell response (41). In addition, VISTA blockade or deficiency of preferentially activated CD8+ T cells, leading to secretion of IFN-γ may influence Th2 cell proliferation. VISTA was demonstrated to negatively inhibit the production of allergen-specific Th2 cells and Th2-mediated antibody secretion (42).

Like most other NCRs, VISTA is highly expressed in tumor-infiltrating Tregs and could suppress tumor-specific immunity. VISTA may also be involved in the differentiation and suppressive function of Treg, both as a receptor on T cells and as a ligand expressed by Tregs. Researchers have found that VISTA can induce and differentiate human and murine Treg cells (2). A reduced ability to convert T cells into iTregs is observed in mice lacking VISTA, but naturally occurring Tregs (nTregs) generation is unaffected (41). Moreover, VISTA blockade decreases the generation of tumor-specific Treg and reverses Treg-mediate suppression (16). However, VISTA blockade also increases T cell proliferation even in the absence of Tregs, indicating that there are other pathways that may involve in Treg mediated suppression (43).

## Myeloid compartments

Human and mouse CD11b^Hi^ myeloid cells, including monocytes, macrophages, granulocytes, and myeloid dendritic cells, express VISTA at high levels; it is conceivable that VISTA expression may also correlate with an immune response (14, 42, 44). In various mouse models of inflammation, such as psoriasis (39), antibody-induced arthritis (45), experimental asthma (46), and lupus nephritis (47), VISTA deficient mice exhibit increased proinflammatory cytokines derived from T cells and myeloid compartments. The transient overexpression of VISTA in human monocytes increases cytokine secretion on protein level and mRNA, such as IL-1β, IL-6, IL-8, and TNFα (44, 48). VISTA is highly expressed by monocytes isolated from chronically HIV-positive individuals and elevated levels of the same cytokines (44). Deleting the cytoplasmic domain of VISTA abolishes this activity, suggesting that VISTA regulates the activation of myeloid cells (44). Further research is needed to explore VISTA’s downstream signaling role in myeloid cells.

Additionally, VISTA is a *P53* regulated surface receptor, expressed on both macrophages and neutrophils, and is essential for dead cell clearance (12). Neutralizing VISTA on macrophages reduced neutrophil efferocytosis *in vitro* (49). In a murine model of bacterial pneumonia, the distribution of VISTA is redirected from the macrophage-neutrophil interaction site to a more disorganized, diffuse surface expression around the macrophage cell and influences the efferocytosis process (49). Additionally, VISTA regulates macrophage functions, influences the M1 (classical) and M2 (alternative) reprogramming of macrophages, and controls the magnitude of innate inflammation *in vivo* (50). Mohamed et al. found that VISTA agonists promote the production of IL-10, miR-221, A20, immune response gene 1 (IRG1), and MerTK, which are involved in both M2 polarization and LPS tolerance, and reduced mediators of M1 polarization, including IFN regulatory factor 5 (IRF5) and IRF8, at transcriptional and protein levels (50). In addition, Nzeteu et al. found that activated M2 macrophages release significantly more VISTA than activated M1 macrophages (51).

Intriguingly, VISTA deficiency did not lead to a severe systemic autoimmune disorder, indicating that other immunoregulatory molecular, such as PD-1, CTLA-4, B7-H3, and B7-H4, might compensate for the VISTA genetic deficiency and mitigated the development of inflammation and autoimmunity induced by genetic loss of VISTA (5).

## VISTA in allergic, autoimmune disease and transplant rejection

Recent evidence suggests that VISTA is also closely associated with allergic reactions, autoimmune diseases, and transplant rejection *via* multiple inhibitory mechanisms, which will be discussed in the following (**Table 1**). It is noteworthy that VISTA is also highly expressed in placental tissues (54). Normal pregnancies prefer a relatively immunosuppressive environment to tolerate fatal antigens, and the acidic environment caused by mild hypoxia may enhance the immunosuppressive activity of VISTA (54, 55). Therefore, VISTA in decidual immune cells can regulate maternal-fetal immunity (54). Research on VISTA’s role in maternal-fetal tolerance is also needed.

**Table 1:** The functions of VISTA in allergic, autoimmune disease and transplantation.

Diseases	Antibody	Role of VISTA	Study(year)	Reference
Allergy				
Skin inflammation	MIH63(blocking)	VISTA suppress the proliferation of, and cytokine production by, T cells.	T Ohno et al. (2017)	(8)
Allergic dermatitis	–	VISTA-Ig significantly suppressed AD symptoms by regulating the abnormal activation of T cells response.	Guo etal. (2021)	(52)
Asthma	4C11(agonistic)	VISTA deficiency promte production of Th2 cytokines (IL-5 and IL-13) and decrease Treg level	Liu et al. (2018)	(46)
Asthma	MIH63(blocking)	VISTA is involved in the regulation of Th 2 cell generation and Th 2 cell-mediated antibody production	T Ohno et al. (2018)	(42)
Autoimmune diseases				
Lupus	MH5A(agonistic)	VISTA on both T cells and myeloid cells could transmit inhibitory signals, resulting in reduces cutaneous disease, autoantibodies, inflammatory cytokines, chemokines, and immune cell expansion.	Han et al.(2019)	(7)
Lupus	13F3(blocking)	VISTA deficiency exacerbates disease of systemic lupus erythematosus and led to increase in renal inflammatory myeloid cells.	Sergent et al. (2017)	(48)
EAE	13F3	VISTA is a negative checkpoint regulator supress T-cell activation, allowing for an enhanced proinflammatory phenotype	Wang et al. (2014)	(35)
Arthritis	8G8(blocking)	VISTA deficiency reduced MMP-3 expression, C5a receptor ecpression, and joint inflammation and tissue damage.	Ceeraz et al. (2017)	(45)
Psoriasis	–	VISTA deficiency led to the hyper-activation of Erk1/2 and Jnk1/2, and augmented the production of IL-23 and IL-17A, and exacerbated psoriasiform inflammation	Li et al (2017)	(40)
Transplantation				
GVHD	MH5A(agonistic)	VISTA as a co-inhibitory receptor on allo-reactive T cell and VISTA on donor T cells is crucial for regulating alloantigen responses.	Flies et al (2011)	(10)
Comeal allografts	MIH 63(blocking)	VISTA-mediated immunomodulation protects the corneal allograft from rejection and promotes the survival of the comeal allograft.	Tomoyuki et al. (2019)	(9)
Viral Infection				
HIV	–	HIV-infected subjects show increased expression of VISTA, which correlated with increased cytokine mRNA expression such as IL-1β, IL-6, IL-8, IL-10, and TNF-α, and immune activation	Bharaj et al. (2014)	(44)
COVID-19	803(agonistic)	Anti-VISTA agonists may suppress the COVID-19 inflammatory signature, as the VISTA agonist downregulated almost 40% of COVID-19 hallmark immune genes.	EiTanbouly at al. (2020)	(53)

## VISTA in allergic diseases

VISTA deficiency and blocking led to a significant increase in infiltrating inflammation in the airways with massive eosinophils in the experimental asthma model induced by OVA (46). VISTA KO mice exhibit increased amounts of Th2-type cytokines, including IL-5 and IL-13, as well as innate inflammatory cytokines, such as MCP-1, TNF-α, and IL-6. VISTA KO mice show a significant reduction in pulmonary CD4+ Foxp3+ Tregs during asthma induction. In addition, VSITA agonist mAb 4C11 suppressed pulmonary inflammation (46). Also, another VISTA antagonist (MIH63) increased Th2-type cytokine production in mice (42). Treatment with the VISTA antagonist (MIH63) during allergen sensitization elevated the IL-13 expression but did not increase the number of eosinophils and asthmatic responses. In contrast, MIH63 treatment during allergen challenge increased IL-4, IL-5, and IL-13 production and accelerated asthmatic responses (42). The results indicate that VISTA is crucial for modulating asthmatic responses (42, 46).

Sibaud et al. found that patients with melanoma given anti-PD-1/PD-L1 therapy were more likely to develop multiple dermatological complications, suggesting that immune checkpoint inhibitors may play important roles in the regulation of skin inflammation and autoimmunity (56). Consistent with this, one line of evidence has shown that VISTA is important in the maintenance of skin homeostasis and inflammation (52). It has been demonstrated that anti-VISTA mAbs can effectively inhibit allergic airway disease and allergic dermatitis (8, 46). T Ohno et al. analyzed the roles of VISTA in allergic skin inflammation using a murine 2,4-dinitro-1-fluorobenzene-induced contact hypersensitivity model. It was found that treatment with anti-mouse VISTA mAb (MIH63) (52, 57) at sensitization, but not at challenge, significantly boosted ear swelling and elevated IFN-γ and TNF-α secretion (52). Further analysis of draining lymph nodes revealed that VISTA antagonist treatment greatly increased the proportions of CD8^+^ CD25^+^ T cells, CD4^+^ IFN-γ^+^ T cells, and CD8^+^ IFN-γ^+^ T cells, and further increased both CD44^high^ fractions in CD8^+^ T cells, but not affect the proportion of Foxp3^+^ Tregs (43, 52). Guo et al. found that VISTA-Ig can successfully alleviate erythema, a horny substance of allergic dermatitis mice (8). As a result, fewer inflammatory cells can be infiltrated, and inflammatory cytokines and IgE are produced (8).

It has been reported that human VISTA-Ig inhibits the proliferation of T cells and reduces cytokine production in T cells (16). However, the detailed role of VISTA in human skin diseases remains to be uncovered. Furthermore, the treatment with VISTA antagonist during the sensitization and challenge stage shows opposite results in the experimental asthma model, and the allergic skin inflammation model still needs further exploration. Accordingly, these observation raises the possibility that the administration of VISTA agonistics could be beneficial in steroid-resistant asthmatic patients and allergic dermatitis patients.

## VISTA in autoinflammation and autoimmunity diseases

Increasingly studies have focused on the relationship between VISTA expressions and autoimmune diseases, such as SLE and RA. It has been discovered that VISTA deletion causes spontaneous autoimmunity and clinically obvious organ-specific autoimmune disease (7). Our discussion will focus on the role of VISTA in autoinflammation and autoimmunity disease, which may subsequently facilitate establishing the rationale for therapeutically enhancing VISTA-mediated pathways to benefit the treatment of multiple autoinflammatory and autoimmune disorders.

## Systemic lupus erythematosus

Sabrina et al. found that patients with systemic lupus erythematosus and discoid lupus erythematosus (DLE) and autoimmune-prone MRL/lpr mice have up-regulated VISTA expression (58). They interbred VISTA-deficient mice with Sle1.Sle3 mice, and analyzed the impact of VISTA deficiency on lupus development in that lupus-prone mouse strain. They found that Sle1.Sle3 VISTA−/− mice showed enhanced CD4+ T cells and myeloid compartment activation and increased secretion of proinflammatory cytokines, chemokines, and interferon (IFN)–regulated genes associated with SLE, including IFNα, IFNγ, tumor necrosis factor, interleukin-10, and CXCL10 (58). A feature of lupus lesions from VISTA KO mice is pDC clustering, and neutrophils infiltrate the skin before clinically evident disease, and subsequently develop more severe systemic lupus erythematosus and inflammatory arthritis (48). An agonistic VISTA antibody can consistently inhibit T cell, pDC, and neutrophil function, suppressing autoimmune lupus in MRL/lpr mice (7). Meanwhile, the treatment with a VSITA-blocking antibody (13F3) exacerbates the progression of lupus and increases amounts of inflammatory myeloid cells and activated T cells (48). Notably, no difference was observed in total anti-dsDNA antibodies, anti-dsDNA-IgG antibody titers, dsDNA-IgM antibodies, or IgG, IgM deposition in the kidney, suggesting that VISTA has little effect on B cells. These findings suggest that VISTA activation could effectively treat systemic lupus erythematosus.

## Rheumatoid arthritis

SLE was exacerbated by deleting VISTA, and VISTA agonist alleviated autoimmune lupus. However, different disease models exhibit opposite effects. For example, rheumatoid arthritis is also a chronic autoimmune inflammation that destroys cartilage, tendon, and bone in particular joints (56, 59). VISTA is expressed in the synovial tissue of joints in healthy and RA patients. By contrast, in a mouse model of collagen antibody-induced arthritis (CAIA), Ceeraz et al. (45) found that both VISTA gene knockout and specific VISTA-blocking antibody (8G8) modulated macrophage responses to immune complexes, significantly induced resistance to arthritis in mice and reduced arthritic inflammation. In addition, it was demonstrated that VISTA deficiency impaired responses of myeloid cells to C5a and regulated macrophage reactions to simulated immune complexes (45). Researchers speculated that it might be related to the type of mouse model and monoclonal antibody used in this study, as well as the type of immune cells involved in the occurrence of RA and, as in another case of CAIA, anti-VISTA antibody (MH5A) greatly reduced arthritic symptoms and joint damage (10). Therefore, future studies may consider selecting other RA models and mAb to further clarify the role of VISTA in the occurrence and development of RA.

## Psoriasis

VISTA also has an inhibitory effect on the occurrence and development of psoriasis. In the imiquimod (IMQ)-induced psoriasis model, VISTA deficiency enhanced the production of IL17 by both γδ T cells and CD4+ Th17 cells and suppressed IMQ-induced TLR7 signaling and IL-23 production, resulting in exacerbated psoriasiform inflammation (40). These results reveal the anti-inflammatory role of VISTA through regulating the IL-23/IL-17 inflammatory axis, leading to the elevated level of inflammatory mediators in the skin and serum, including IL-1β, IL-6, IL-17A, IL-22, IL-23, IFN-γ, TNF-α, and CXCL2 (40).

## VISTA and allogeneic transplantation

Organ transplantation is still challenging in clinical practice due to allogenic transplant rejection and Graft versus host disease. Therefore, numerous researchers are testing the clinical feasibility of targeting immunomodulatory molecules to prevent GVHD and prolong organ transplant survival.

GVHD is a reaction of activated allogeneic or major histocompatibility complex (MHC) mismatched T cells directed against host tissues, which may result in wide tissue damage, increasing the risk of metabolic disorder, opportunistic infection, and malignancy, and generally require immune suppression (11, 60). GVHD is commonly found in allogeneic hematopoietic cell transplantation for treating hematologic malignancies and in the setting of solid organ transplantation. Flies et al. found that VISTA^-/-^ T cells induce exacerbated graft-versus-host disease compared to wild-type T cells and found the immunosuppressive impact of VISTA agonists in acute GVHD (11). By immunohistochemistry analysis, they found that the VISTA agonists (MH5A) can greatly reduce infiltrating T cells and suppress T cell activation in all GVHD target organs, such as the liver, spleen, kidney, and lung (2, 11). In addition, the VISTA agonists targeting VISTA on donor T cells can prevent GVHD (10).

Tomoyuki et al. (9) examined the role of VISTA in the immune-privilege status of corneal allotransplantation and allospecific anterior chamber-associated immune deviation (ACAID). The researchers found that VISTA-mediated immunomodulation protects corneal allografts from rejection and promotes survival. Two possible mechanisms are explored in this study. One was that VISTA is involved in the induction of donor-specific ACAID, that antigens-specific systemic immune tolerance to eye-derived antigens *via* splenic CD8^+^ CD103^+^ T regulatory cells (9). Additionally, they found that the corneal VISTA mediates immune suppression and reduces inflammatory cells within the eyes.

VISTA may contribute to GVHD and allograft rejection, and forced expression of VISTA may be a potential therapeutic strategy for conferring an immune-privileged status to suppress GVHD allograft rejection. However, the precise mechanism of VISTA in the modulation of T-cell response remains elucidated. Therefore, studies are needed to determine the signaling pathways mediated by VISTA on APCs and T cells, which will facilitate manipulation of the immune system for maintaining tolerance and graft survival with the least side effects.

## VISTA in other CNS inflammation and diseases

Microglia, a major myeloid cell in the central nervous system (CNS). VISTA is most abundantly expressed by mouse and human microglia and involved in the immune response of microglia (43, 61). Therefore, understanding the role of VISTA in CNS inflammation and diseases is important. In accordance with SLE, anti-VISTA Ab administration *in vivo* enhanced disease progression in a passive EAE model. Furthermore, VISTA-deficient mice significantly enhanced the development and progression of EAE on a disease-prone transgenic background, suggesting that VISTA deficiency overcame additional immune-regulatory mechanisms, leading to elevated activated encephalitogenic T cells in the periphery and promoting its infiltration into the central nervous system (35).

As the VISTA may be involved in immune surveillance and uptake of apoptotic neurons or other debris by microglia (12, 61). Borggrewe et al. (62) summarized the role of VISTA in CNS inflammation, aging, and neurodegenerative diseases. The expression of VISTA by microglia is consistently decreased in neurodegenerative diseases (NDD), including Alzheimer’s disease, frontotemporal dementia, Parkinson’s disease, and amyotrophic lateral sclerosis. Reduction in VISTA expression during NDD leads to elevated production of cytokines and inhibits the clearance of cell debris which might enhance neuroinflammation (12, 40, 61).

VISTA expression consistently decreases in chronic active multiple sclerosis (MS) lesions and MS mouse model (61). Microglia VISTA slightly decreases in MS white matter, whereas there is no significant difference in MS grey matter (63, 64). The loss of VISTA expression on microglia in MS may exacerbate the activation of infiltrating T cells in the lesion, further enhancing inflammation and tissue destruction (65). Microglia also involves various CNS diseases, including stroke and Purkinje cell degeneration. The expression of VISTA by microglia is reduced 2-fold in the stroke murine model (62). Although inhibiting the activation of microglia during stroke facilitate to produce beneficial outcomes, microglial activation is also critical to counteract neuronal death and enhance neurogenesis and angiogenesis (62, 66).

Alzheimer’s and Parkinson’s disease may develop due to reduced VISTA expression in microglia during aging. For example, in mice, VISTA expression is reduced in microglia in aged mice compared with younger mice. And individuals > 50 years of age show a slight increase in VISTA expression compared with those < 50 years (62).

## VISTA and viral infection

VISTA could also impact human immunodeficiency virus (HIV)-induced immune activation and T-cell response. Monocytes from HIV-infected subjects show increased expression of VISTA, which correlated with increased cytokine mRNA expression such as IL-1β, IL-6, IL-8, IL-10, and TNF-α, and immune activation (44), and these results indicated that VISTA might play a critical role in regulating the immune response in HIV infection. Additionally, VISTA may induce phagocytosis of HIV-infected T cells and subsequent upregulation of apoptosis (67). VISTA overexpression on both phagocytes and HIV-infected CEM-SS T cells facilitated phagocytosis (66). Moreover, it has been suggested that CXCL10 might serve as a prognostic biomarker and an essential pathogenic mediator of COVID-19 in recent studies (68, 69). The potential immunoregulatory role of VISTA in COVID-induced inflammation has also been explored as the anti-VISTA mAb suppresses CXCL10. Enrichment analysis of the COVID-19 immune profile indicates that anti-VISTA agonists may suppress the COVID-19 inflammatory signature, as the VISTA agonist downregulated almost 40% of COVID-19 hallmark immune genes (53). However, The underlining mechanism is still unknown, which could be related to VISTA agonist’s reduction of FcgRIIIa, as the Fc receptor responses have been reported as an immunopathologic feature of COVID-19 infection (53). The impact of VISTA on myeloid chemotaxis also makes it a promising target in COVID-19 cytokine storm management. In addition, heightened cytokine production from myeloid cells likely contributes to cytokine-release syndrome (CRS) induced by CAR-T cell therapy, and VISTA agonistic may ameliorate the innate inflammation of the CAR-T-induced CRS (53). Meanwhile, VISTA agonists likely would exert minimal inhibitory impact on activated CAR-T cells directly (53). However, there are limited numbers of studies on viral infection and VISTA biology, which topic remains largely unexplored.

## Targeting VISTA in autoimmunity

Multiple functional outcomes of VISTA modulation are plausible in autoimmune diseases. VISTA antibodies can be divided into agonistic or antagonistic antibodies according to their function. VISTA agonistics has been shown to limit immune response and suppress proinflammatory cytokine production in several mice autoimmune diseases models, such as EAE, murine lupus nephritis, K/BxN arthritis, IMQ-induced psoriasis, and GVHD (5, 10). VISTA agonist mAb, such as MH5A (7, 10, 11) and 4C11 (46). Using these VISTA antagonistic in mice, such as 13F3 (14, 43), and MIH63 (42) exacerbates the severity of the allergy and autoimmune diseases, including asthma and EAE. On the contrary, VISTA antagonistic 8G8 suppresses the collagen antibody-induced arthritis (45). Researchers have no consensus regarding the exact function of the MH5A clone (70). MH5A clone acts as an agonist in the GVHD model and autoimmune lupus in MRL/lpr mice, whereas in the case of CAIA, Ceeraz et al. used it as an antagonist to reduce joint inflammation (45). The exact epitopes and properties involved with different monoclonal antibody against VISTA may account for these apparent various results (71).

Importantly, VISTA may also be involved in a large variety of functions, including phagocytosis, cytokine response, and chemotaxis, which make it complex to predict the outcome of VISTA modulation. Moreover, systemically using anti-VISTA antibodies may impact the immune function unpredictably. Therefore, it urges us to explore precise therapeutic antibodies which target distinct epitopes. In addition, an innovative strategy for engineering pH-selective anti-VISTA antibodies has been developed, targeting only the PH- dysregulation sites.

## Conclusions

VISTA is a critical immune-checkpoint regulator that maintains peripheral tolerance and controls autoimmunity. Recent advances in targeting VISTA have shown promising results in immune-related diseases such as cancer and autoimmunity. VISTA-Ig and genetic VISTA ablation promotes proinflammatory cytokine production and leads to a susceptible condition under which autoimmune diseases are more likely to develop. VISTA manipulation or targeting to enhance or suppress its activity may offer promising therapeutic approaches for allergic, autoimmune disease and transplantation. The knowledge gap concerning VISTA binding partners is a critical limiting step in developing targeted therapy. Identifying VISTA’s binding partner is critical for clinical benefits and will further offer insight into the cellular and molecular immunomodulatory mechanisms of VISTA signaling. An in-depth understanding of VISTA’s immunomodulatory mechanisms has provided exceptional opportunities to therapeutically target or manipulate VISTA and design synergistic combination therapies for tolerance maintenance in allergic and autoimmune diseases and organ transplants.

## Author contributions

All authors listed have made a substantial, direct, and intellectual contribution to the work and approved it for publication.

## References

[1] Chen L Flies DB. Molecular mechanisms of T cell co-stimulation and co-inhibition. Nat Rev Immunol (2013) 13:227–42. doi: 10.1038/nri3405 PMC3786574 23470321

[2] Flies DB Han X Higuchi T Zheng L Sun J Ye JJ. Coinhibitory receptor PD-1H preferentially suppresses CD4(+) T cell-mediated immunity. J Clin Invest (2014) 124:1966–75. doi: 10.1172/JCI74589 PMC4001557 24743150

[3] Azuma M. Co-Signal molecules in T-cell activation : historical overview and perspective. Adv Exp Med Biol (2019) 1189:3–23. doi: 10.1007/978-981-32-9717-3_1 31758529

[4] Nowak EC Lines JL Deng J Sarde A Mabaera R. Immunoregulatory functions of VISTA. Immunol Rev (2017) 276:66–79. doi: 10.1111/imr.12525 28258694 PMC5702497

[5] ElTanbouly MA Zhao Y Nowak E Li J Schaafsma E Le Mercier I. VISTA is a checkpoint regulator for naive T cell quiescence and peripheral tolerance. Science (2020) 367(6475):eaay0524. doi: 10.1126/science.aay0524 31949051 PMC7391053

[6] Guo Y Wang AY. Novel immune check-point regulators in tolerance maintenance. Front Immunol (2015) 6:421. doi: 10.3389/fimmu.2015.00421 26347744 PMC4539525

[7] Han X Vesely MD Yang W Sanmamed MF Badri T Alawa J. PD-1H (VISTA)-mediated suppression of autoimmunity in systemic and cutaneous lupus erythematosus. Sci Transl Med (2019) 11(522):eaax1159. doi: 10.1126/scitranslmed.aax1159 31826980

[8] Guo Y Ding C Chang T Yuan W Liu X. VISTA-ig ameliorates OXA-induced allergic dermatitis symptoms in mice. Immunopharmacol Immunotoxicol (2021) 43:380–5. doi: 10.1080/08923973.2021.1925907 34028330

[9] Kunishige T Taniguchi H Ohno T Azuma M Hori J. VISTA is crucial for corneal allograft survival and maintenance of immune privilege. Invest Ophthalmol Vis Sci (2019) 60:4958–65. doi: 10.1167/iovs.19-27322 31790558

[10] Flies DB Wang S Xu H Chen L. Cutting edge: a monoclonal antibody specific for the programmed death-1 homolog prevents graft-versus-host disease in mouse models. J Immunol (2011) 187:1537–41. doi: 10.4049/jimmunol.1100660 PMC3150865 21768399

[11] Flies DB Higuchi T Chen L. Mechanistic assessment of PD-1H coinhibitory receptor-induced T cell tolerance to allogeneic antigens. J Immunol (2015) 194:5294–304. doi: 10.4049/jimmunol.1402648 PMC4433880 25917101

[12] Yoon KW Byun S Kwon E Hwang SY Chu K Hiraki M. Control of signaling-mediated clearance of apoptotic cells by the tumor suppressor p53. Science (2015) 349:1261669. doi: 10.1126/science.1261669 26228159 PMC5215039

[13] Aloia L Parisi S Fusco L Pastore L Russo T. Differentiation of embryonic stem cells 1 (Dies1) is a component of bone morphogenetic protein 4 (BMP4) signaling pathway required for proper differentiation of mouse embryonic stem cells. J Biol Chem (2010) 285:7776–83. doi: 10.1074/jbc.M109.077156 PMC2844221 20042595

[14] Wang L Rubinstein R Lines JL Wasiuk A Ahonen C Guo Y. VISTA, a novel mouse ig superfamily ligand that negatively regulates T cell responses. J Exp Med (2011) 208:577–92. doi: 10.1084/jem.20100619 PMC3058578 21383057

[15] Mehta N Maddineni S Mathews II Andres Parra Sperberg R Huang PS Cochran JR. Structure and functional binding epitope of V-domain ig suppressor of T cell activation. Cell Rep (2019) 28:2509–2516 e2505. doi: 10.1016/j.celrep.2019.07.073 31484064

[16] Lines JL Pantazi E Mak J Sempere LF Wang L O'Connell S. VISTA is an immune checkpoint molecule for human T cells. Cancer Res (2014) 74:1924–32. doi: 10.1158/0008-5472.CAN-13-1504 PMC3979527 24691993

[17] ElTanbouly MA Croteau W Noelle RJ Lines JL. VISTA: a novel immunotherapy target for normalizing innate and adaptive immunity. Semin Immunol (2019) 42:101308. doi: 10.1016/j.smim.2019.101308 31604531 PMC7233310

[18] Mulati K Hamanishi J Matsumura N Chamoto K Mise N Abiko K. VISTA expressed in tumour cells regulates T cell function. Br J Cancer (2019) 120:115–27. doi: 10.1038/s41416-018-0313-5 PMC6325144 30382166

[19] Gao J Ward JF Pettaway CA Shi LZ Subudhi SK Vence LM. VISTA is an inhibitory immune checkpoint that is increased after ipilimumab therapy in patients with prostate cancer. Nat Med (2017) 23:551–5. doi: 10.1038/nm.4308 PMC5466900 28346412

[20] Zhang M Pang HJ Zhao W Li YF Yan LX Dong ZY. VISTA expression associated with CD8 confers a favorable immune microenvironment and better overall survival in hepatocellular carcinoma. BMC Cancer (2018) 18:511. doi: 10.1186/s12885-018-4435-1 29720116 PMC5932869

[21] Blando J Sharma A Higa MG Zhao H Vence L Yadav SS. Comparison of immune infiltrates in melanoma and pancreatic cancer highlights VISTA as a potential target in pancreatic cancer. Proc Natl Acad Sci USA (2019) 116:1692–7. doi: 10.1073/pnas.1811067116 PMC6358697 30635425

[22] Boger C Behrens HM Kruger S Rocken C. The novel negative checkpoint regulator VISTA is expressed in gastric carcinoma and associated with PD-L1/PD-1: a future perspective for a combined gastric cancer therapy? Oncoimmunology (2017) 6:e1293215. doi: 10.1080/2162402X.2017.1293215 28507801 PMC5414883

[23] Xie S Huang J Qiao Q Zang W Hong S Tan H. Expression of the inhibitory B7 family molecule VISTA in human colorectal carcinoma tumors. Cancer Immunol Immunother (2018) 67:1685–94. doi: 10.1007/s00262-018-2227-8 PMC11028359 30128738

[24] Wu L Deng WW Huang CF Bu LL Yu GT Mao L. Expression of VISTA correlated with immunosuppression and synergized with CD8 to predict survival in human oral squamous cell carcinoma. Cancer Immunol Immunother (2017) 66:627–36. doi: 10.1007/s00262-017-1968-0 PMC11028774 28236118

[25] Xu W Dong J Zheng Y Zhou J Yuan Y Ta HM. Immune-checkpoint protein VISTA regulates antitumor immunity by controlling myeloid cell-mediated inflammation and immunosuppression. Cancer Immunol Res (2019) 7:1497–510. doi: 10.1158/2326-6066.CIR-18-0489 PMC6726548 31340983

[26] Wang J Wu G Manick B Hernandez V Renelt M Erickson C. VSIG-3 as a ligand of VISTA inhibits human T-cell function. Immunology (2019) 156:74–85. doi: 10.1111/imm.13001 30220083 PMC6283650

[27] Johnston RJ Su LJ Pinckney J Critton D Boyer E Krishnakumar A. VISTA is an acidic pH-selective ligand for PSGL-1. Nature (2019) 574:565–70. doi: 10.1038/s41586-019-1674-5 31645726

[28] Yuan L Tatineni J Mahoney KM Freeman GJ. VISTA: a mediator of quiescence and a promising target in cancer immunotherapy. Trends Immunol (2021) 42:209–27. doi: 10.1016/j.it.2020.12.008 PMC8088836 33495077

[29] Laszik Z Jansen PJ Cummings RD Tedder TF McEver RP Moore KL. P-selectin glycoprotein ligand-1 is broadly expressed in cells of myeloid, lymphoid, and dendritic lineage and in some nonhematopoietic cells. Blood (1996) 88:3010–21. doi: 10.1182/blood.V88.8.3010.bloodjournal8883010 8874199

[30] Suzu S Hayashi Y Harumi T Nomaguchi K Yamada M Hayasawa H. Molecular cloning of a novel immunoglobulin superfamily gene preferentially expressed by brain and testis. Biochem Biophys Res Commun (2002) 296(5):1215–21. doi: 10.1016/s0006-291x(02)02025-9 12207903

[31] Pouyani T Seed B. PSGL-1 recognition of p-selectin is controlled by a tyrosine sulfation consensus at the PSGL-1 amino terminus. Cell (1995) 83(2):333–43. doi: 10.1016/0092-8674(95)90174-4 7585950

[32] Vachino G Chang XJ Veldman GM Kumar R Sako D Fouser LA. Cumming DA. p-selectin glycoprotein ligand-1 is the major counter-receptor for p-selectin on stimulated T cells and is widely distributed in non-functional form on many lymphocytic cells. J Biol Chem (1995) 270(37):21966–74. doi: 10.1074/jbc.270.37.21966 7545173

[33] Gabr MT Gambhir SS. Discovery and optimization of small-molecule ligands for V-domain ig suppressor of T-cell activation (VISTA). J Am Chem Soc (2020) 142:16194–8. doi: 10.1021/jacs.0c07276 32894020

[34] Yasinska IM Meyer NH Schlichtner S Hussain R Siligardi G Casely-Hayford M. Ligand-receptor interactions of galectin-9 and VISTA suppress human T lymphocyte cytotoxic activity. Front Immunol (2020) 11:580557. doi: 10.3389/fimmu.2020.580557 33329552 PMC7715031

[35] Wang L Le Mercier I Putra J Chen W Liu J Schenk AD. Disruption of the immune-checkpoint VISTA gene imparts a proinflammatory phenotype with predisposition to the development of autoimmunity. Proc Natl Acad Sci USA (2014) 111:14846–51. doi: 10.1073/pnas.1407447111 PMC4205642 25267631

[36] Rodriguez-Manzanet R Sanjuan MA Wu HY Quintana FJ Xiao S Anderson AC. T And b cell hyperactivity and autoimmunity associated with niche-specific defects in apoptotic body clearance in TIM-4-deficient mice. Proc Natl Acad Sci USA (2010) 107:8706–11. doi: 10.1073/pnas.0910359107 PMC2889349 20368430

[37] Feldman CH Hiraki LT Liu J Fischer MA Solomon DH Alarcón GS. Epidemiology and sociodemographics of systemic lupus erythematosus and lupus nephritis among US adults with Medicaid coverage, 2000-2004. Arthritis Rheum (2013) 65:753–63. doi: 10.1002/art.37795 PMC3733212 23203603

[38] Hid Cadena R Reitsema RD Huitema MG van Sleen Y van der Geest KSM Heeringa P. Decreased expression of negative immune checkpoint VISTA by CD4+ T cells facilitates T helper 1, T helper 17, and T follicular helper lineage differentiation in GCA. Front Immunol (2019) 10:1638. doi: 10.3389/fimmu.2019.01638 31379838 PMC6646729

[39] Gaffen SL Jain R Garg AV Cua DJ. The IL-23-IL-17 immune axis: from mechanisms to therapeutic testing. Nat Rev Immunol (2014) 14:585–600. doi: 10.1038/nri3707 25145755 PMC4281037

[40] Li N Xu W Yuan Y Ayithan N Imai Y Wu X. Immune-checkpoint protein VISTA critically regulates the IL-23/IL-17 inflammatory axis. Sci Rep (2017) 7:1485. doi: 10.1038/s41598-017-01411-1 28469254 PMC5431161

[41] Wang Q He J Flies DB Luo L Chen L. Programmed death one homolog maintains the pool size of regulatory T cells by promoting their differentiation and stability. Sci Rep (2017) 7:6086. doi: 10.1038/s41598-017-06410-w 28729608 PMC5519767

[42] Ohno T Zhang C Kondo Y Kang S Furusawa E Tsuchiya K. The immune checkpoint molecule VISTA regulates allergen-specific Th2-mediated immune responses. Int Immunol (2018) 30:3–11. doi: 10.1093/intimm/dxx070 29267882

[43] Le Mercier I Chen W Lines JL Day M Li J Sergent P. VISTA regulates the development of protective antitumor immunity. Cancer Res (2014) 74:1933–44. doi: 10.1158/0008-5472.CAN-13-1506 PMC4116689 24691994

[44] Bharaj P Chahar HS Alozie OK Rodarte L Bansal A Goepfert PA. Characterization of programmed death-1 homologue-1 (PD-1H) expression and function in normal and HIV infected individuals. PloS One (2014) 9:e109103. doi: 10.1371/journal.pone.0109103 25279955 PMC4184823

[45] Ceeraz S Eszterhas SK Sergent PA Armstrong DA Ashare A Broughton T. VISTA deficiency attenuates antibody-induced arthritis and alters macrophage gene expression in response to simulated immune complexes. Arthritis Res Ther (2017) 19:270. doi: 10.1186/s13075-017-1474-y 29216931 PMC5721690

[46] Liu H Li X Hu L Zhu M He B Luo L. A crucial role of the PD-1H coinhibitory receptor in suppressing experimental asthma. Cell Mol Immunol (2018) 15:838–45. doi: 10.1038/cmi.2017.16 PMC6203798 28479600

[47] Ceeraz S Sergent PA Plummer SF Schned AR Pechenick D Burns CM. VISTA deficiency accelerates the development of fatal murine lupus nephritis. Arthritis Rheumatol (2017) 69(4):814–25. doi: 10.1002/art.40020 PMC5723399 27992697

[48] Sergent PA Plummer SF Pettus J Mabaera R DeLong JK Pechenick DA. Blocking the VISTA pathway enhances disease progression in (NZB × NZW) F1 female mice. Lupus (2018) 27(2):210–6. doi: 10.1177/0961203317716322 PMC5753845 28659048

[49] Cohen TS Jones-Nelson O Hotz M Cheng L Miller LS Suzich J. S. aureus blocks efferocytosis of neutrophils by macrophages through the activity of its virulence factor alpha toxin. Sci Rep (2016) 6:35466. doi: 10.1038/srep35466 27739519 PMC5064327

[50] ElTanbouly MA Schaafsma E Smits NC Shah P Cheng C Burns C. VISTA re-programs macrophage biology through the combined regulation of tolerance and anti-inflammatory pathways. Front Immunol (2020) 11:580187. doi: 10.3389/fimmu.2020.580187 33178206 PMC7593571

[51] Noubissi Nzeteu GA Schlichtner S David S Ruppenstein A Fasler-Kan E Raap U. Macrophage differentiation and polarization regulate the release of the immune checkpoint protein V-domain ig suppressor of T cell activation. Front Immunol (2022) 13:837097. doi: 10.3389/fimmu.2022.837097 35634346 PMC9132587

[52] Ohno T Kondo Y Zhang C Kang S Azuma M. Immune checkpoint molecule, VISTA regulates T-Cell-Mediated skin inflammatory responses. J Invest Dermatol (2017) 137:1384–6. doi: 10.1016/j.jid.2016.10.049 28104378

[53] ElTanbouly MA Zhao Y Schaafsma E Burns CM Mabaera R Cheng C. VISTA: a target to manage the innate cytokine storm. Front Immunol (2020) 11:595950. doi: 10.3389/fimmu.2020.595950 33643285 PMC7905033

[54] Zhao SJ Muyayalo KP Luo J Huang D Mor G Liao AH. Next generation of immune checkpoint molecules in maternal-fetal immunity. Immunol Rev (2022) 308:40–54. doi: 10.1111/imr.13073 35234305

[55] Ma LN Huang XB Muyayalo KP Mor G Liao AH. Lactic acid: a novel signaling molecule in early pregnancy? Front Immunol (2020) 11:279. doi: 10.3389/fimmu.2020.00279 32180770 PMC7057764

[56] Sibaud V Meyer N Lamant L Vigarios E Mazieres J Delord JP. Dermatologic complications of anti-PD-1/PD-L1 immune checkpoint antibodies. Curr Opin Oncol (2016) 28(4):254–63. doi: 10.1097/CCO.0000000000000290 27136138

[57] Kondo Y Ohno T Nishii N Harada K Yagita H Azuma M. Differential contribution of three immune checkpoint (VISTA, CTLA-4, PD-1) pathways to antitumor responses against squamous cell carcinoma. Oral Oncol (2016) 57:54–60. doi: 10.1016/j.oraloncology.2016.04.005 27208845

[58] Ceeraz S Sergent PA Plummer SF Schned AR Pechenick D Burns CM. VISTA Deficiency Accelerates the Development of Fatal Murine Lupus Nephritis. Arthritis Rheumatol (2017) 69:814–825. doi: 10.1002/art.40020 PMC5723399 27992697

[59] Firestein GS. Immunologic mechanisms in the pathogenesis of rheumatoid arthritis. J Clin Rheumatol (2005) 11:S39–44. doi: 10.1097/01.rhu.0000166673.34461.33 16357749

[60] Kekre N Antin JH. Emerging drugs for graft-versus-host disease. Expert Opin Emerging Drugs (2016) 21:209–18. doi: 10.1517/14728214.2016.1170117 27007595

[61] Borggrewe M Grit C Den Dunnen WFA Burm SM Bajramovic JJ Noelle RJ. VISTA expression by microglia decreases during inflammation and is differentially regulated in CNS diseases. Glia (2018) 66:2645–58. doi: 10.1002/glia.23517 PMC6585704 30306644

[62] Borggrewe M Kooistra SM Noelle RJ Eggen BJL Laman JD. Exploring the VISTA of microglia: immune checkpoints in CNS inflammation. J Mol Med (Berl) (2020) 98:1415–30. doi: 10.1007/s00109-020-01968-x PMC7525281 32856125

[63] Elkjaer ML Frisch T Reynolds R Kacprowski T Burton M Kruse TA. Molecular signature of different lesion types in the brain white matter of patients with progressive multiple sclerosis. Acta Neuropathol Commun (2019) 7:205. doi: 10.1186/s40478-019-0855-7 31829262 PMC6907342

[64] van der Poel M Ulas T Mizee MR Hsiao CC Miedema SSM Adelia. Transcriptional profiling of human microglia reveals grey-white matter heterogeneity and multiple sclerosis-associated changes. Nat Commun (2019) 10:1139. doi: 10.1038/s41467-019-08976-7 30867424 PMC6416318

[65] Voet S Prinz M van Loo G. Microglia in central nervous system inflammation and multiple sclerosis pathology. Trends Mol Med (2019) 25:112–23. doi: 10.1016/j.molmed.2018.11.005 30578090

[66] Qin C Zhou LQ Ma XT Hu ZW Yang S Chen M. Dual functions of microglia in ischemic stroke. Neurosci Bull (2019) 35:921–33. doi: 10.1007/s12264-019-00388-3 PMC6754485 31062335

[67] Xu X Petersen S Rodriguez C Yi G. VISTA facilitates phagocytic clearance of HIV infected CEM-SS T cells. Heliyon (2021) 7:e07496. doi: 10.1016/j.heliyon.2021.e07496 34401556 PMC8353305

[68] Huang C Wang Y Li X Ren L Zhao J Hu Y. Clinical features of patients infected with 2019 novel coronavirus in wuhan, China. Lancet (2020) 395:497–506. doi: 10.1016/S0140-6736(20)30183-5 31986264 PMC7159299

[69] Mehta P McAuley DF Brown M Sanchez E Tattersall RS Manson JJ. COVID-19: consider cytokine storm syndromes and immunosuppression. Lancet (2020) 395:1033–4. doi: 10.1016/S0140-6736(20)30628-0 PMC7270045 32192578

[70] Molloy M. Trustees of Dartmouth college and immunext Inc. anti-human VISTA antibodies and use thereof.

[71] Mehta N Maddineni S Kelly RL Lee RB Hunter SA Silberstein JL. An engineered antibody binds a distinct epitope and is a potent inhibitor of murine and human VISTA. Sci Rep (2020) 10:15171. doi: 10.1038/s41598-020-71519-4 32938950 PMC7494997

